# Letter to the Editor regarding: ‘Circulating fibroblast growth factor 21 is associated with blood pressure in the Chinese population: a community-based study’

**DOI:** 10.1080/07853890.2025.2575107

**Published:** 2025-10-15

**Authors:** Jianren Wen, Jingxuan Hu, Guohui Zou

**Affiliations:** a Jiangxi University of Chinese Medicine, Nanchang, China; b The Affiliated Hospital of Jiangxi University of Chinese Medicine, Nanchang, China

To the Editor

The article by Zhen et al. offers insights into a significant association between circulating fibroblast growth factor 21 (FGF21) and blood pressure in a community-based Chinese population [1]. Their findings contribute important evidence supporting FGF21 as a potential biomarker for hypertension in Asian cohorts. The rigorous methodology and clear presentation of results are notable. However, several aspects warrant further discussion to strengthen the clinical relevance of these findings.

One limitation that merits consideration is the use of a single measurement of FGF21 levels, which may not accurately reflect long-term exposure due to influences such as fasting status, diurnal variation and metabolic fluctuations [2–4]. Therefore, repeated measurements or averaged levels in future studies could provide a more reliable assessment of the relationship between FGF21 and blood pressure.

Although the study adjusted for several confounders, residual confounding from unmeasured variables, such as dietary sodium intake, psychosocial stress and insulin resistance indices, might affect the observed associations. Incorporating these factors in future analyses would enhance the robustness of the conclusions.

While subgroup analysis by sex was performed, further stratification by age, body mass index or glucose tolerance status could reveal significant heterogeneity in the FGF21–blood pressure relationship [5]. Such analyses may help identify high-risk subgroups that could benefit most from FGF21-targeted screening or intervention. Additionally, as the data were drawn exclusively from Shenzhen, the generalizability of the findings to other Chinese populations with distinct genetic and environmental backgrounds may be limited. External validation using multi-regional cohorts would help confirm the broader applicability of the results.

To address these limitations and advance the field, we propose the integration of methodologies from adjacent disciplines. For instance, employing unsupervised machine learning algorithms could move beyond traditional stratification to identify novel metabo-phenotypic clusters where the FGF21–blood pressure relationship is most pronounced. Furthermore, future multi-regional cohorts could incorporate genetic data (e.g. polymorphisms in FGF21 signalling pathways) and objective measures of dietary intake (e.g. via mobile health technology) to dissect the genetic and environmental determinants of population heterogeneity, thereby translating a generalized association into personalized risk stratification.

In summary, we commend Zhen et al. for their insightful exploration at the intersection of metabolism and cardiovascular health. This study lays a solid foundation for subsequent research aimed at elucidating the mechanistic pathways by which FGF21 regulates blood pressure and systematically evaluating its therapeutic potential.

## Data Availability

Data availability is not applicable to this article as no new data were created or analysed in this study.
